# Comprehensive analysis of the oncogenic and immunological role of FAP and identification of the ceRNA network in human cancers

**DOI:** 10.18632/aging.204707

**Published:** 2023-05-09

**Authors:** Weiqian Mai, Qingyou Liu, Jiasheng Li, Mincheng Zheng, Fuman Yan, Hui Liu, Yuhe Lei, Jinwen Xu, Jiean Xu

**Affiliations:** 1 School of Medicine, School of Life Science and Engineering, Foshan University, Foshan 528000, China; 2 Integrative Medicine Research Center, School of Basic Medical Sciences, Guangzhou University of Chinese Medicine, University Town, Guangzhou 510006, China; 3 Department of Physiology, School of Basic Medical Sciences, Guangzhou University of Chinese Medicine, University Town, Guangzhou 510006, China; 4 Department of Pharmacy, Shenzhen Hospital of Guangzhou University of Chinese Medicine, Shenzhen 518034, China

**Keywords:** fibroblast activation protein-alpha (FAP), prognosis, immunological infiltration, competing endogenous RNA (ceRNA)

## Abstract

Fibroblast activation protein-alpha (FAP) is a transmembrane serine protease involving in tissue remodeling. Previous studies report that FAP is highly expressed in certain tumors and participated in oncogenesis. However, there is still lack of systematic and in-depth analysis of FAP based on clinical big data. Here, we comprehensively map the FAP expression profile, prognostic outcome, genetic alteration, immune infiltration across over 30 types of human cancers through multiple datasets including TCGA, CPTAC, and cBioPortal. We find that FAP is up-regulated in most cancer types, and increased FAP expression is associated with advanced pathological stages or poor prognosis in several cancers. Furthermore, FAP is significantly correlated with the infiltration of cancer-associated fibroblasts, macrophages, myeloid dendritic cells, as well as endothelia cells. Immunosuppressive checkpoint proteins or cytokines expression, microsatellite instability and tumor mutational burden analysis also indicate the regulation role of FAP in tumor progression. Gene enrichment analysis demonstrates that ECM-receptor interaction as well as extracellular matrix and structure process are linked to the potential mechanism of FAP in tumor pathogenesis. The ceRNA network is also constructed and identified the involvement of LINC00707/hsa-miR-30e-5p/FAP, LINC02535/hsa-miR-30e-5p/FAP, LINC02535/hsa-miR-30d-5p/FAP, as well as AC026356.1/hsa-miR-30d-5p/FAP axis in tumor progression. In conclusion, our study offers new insights into the oncogenic and immunological role of FAP from a pan-cancer perspective, providing new clues for developing novel targeted anti-tumor strategies.

## INTRODUCTION

Fibroblast activation protein-alpha (FAP), also known as seprase, is a type II transmembrane serine protease that belongs to the prolyl peptidase family. Within this family, FAP and dipeptidyl peptidase IV (DPPIV) share the highest homology with up to 70% amino acid identity. FAP harbors both dipeptidyl peptidase and endopeptidase activity, while DPPIV possesses only dipeptidyl peptidase activity. When activated, FAP dimerizes and exclusively cleaves the post-proline peptide bond of peptide or protein substrates [1–4]. FAP is scarcely expressed in normal adult tissues. While under pathological circumstance, due to excessively remodeling extracellular matrix and abnormally altering intracellular signaling pathways, FAP is linked to multiple pathologies including liver cirrhosis, pulmonary fibrosis, Crohn’s disease, arthritis, atherosclerosis, and even tumor. High expression of FAP has been reported in certain human malignancies, including myeloma, breast cancer, lung cancer and gastrointestinal carcinoma. Upon several cases, elevated FAP expression is associated with greater tumor size, advanced clinical stage and lymph node metastases [5–10].

Nevertheless, throughout literature search, we find that comprehensive analysis of FAP based on big clinical data is still ill-established, and in-depth research of the relationship between FAP expression and tumor immunological profile is still lacking [11–13]. Thus, in this study, we systematically evaluated the FAP expression profile across thousands of cancer cases based on the TCGA, GTEx and CPTAC datasets, to establish the link between FAP expression and the clinical prognostic features. Aiming to understand the oncogenic and immunological role of FAP in cancer pathogenesis, correlation between FAP expression and genetic mutation, microsatellite instability (MSI), tumor mutational burden (TMB), immunological cells infiltration, immunosuppressive checkpoint proteins or cytokines expression were also explored. Moreover, the gene enrichment analysis as well as the prediction and construction of ceRNA network were also conducted to further figure out the potential mechanism. Our study provides new insights into FAP from a pan-cancer perspective, indicates that FAP may serve as a prognostic biomarker and promising therapeutic target in tumor treatment, and offers new clues for developing novel targeted anti-tumor treatment.

## RESULTS

### Expression of FAP in human cancers

**Figure 1:**
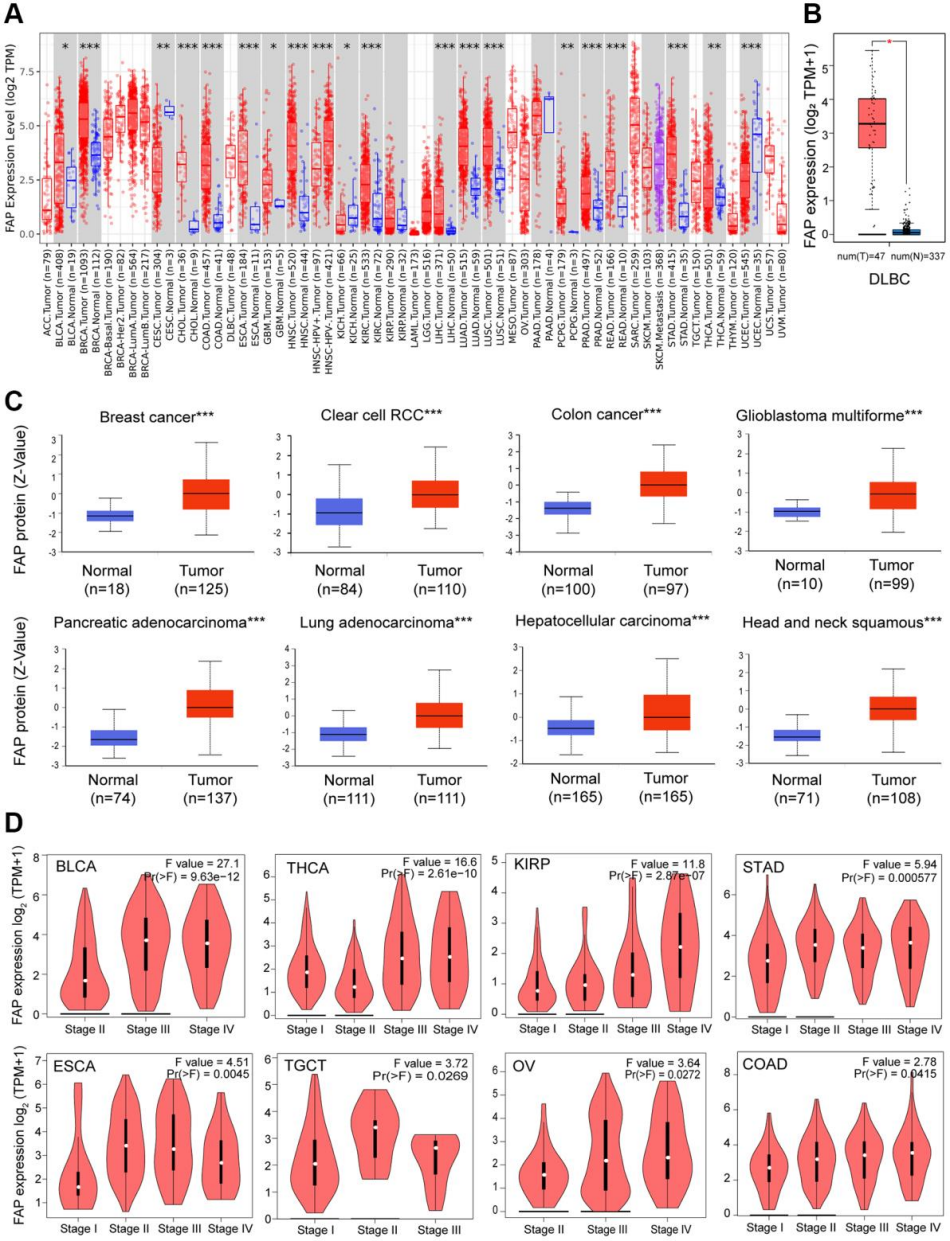
**FAP expression profile in human cancers.** (**A**) Human FAP expression levels in different cancer types from the TCGA database. The statistical significance was computed by the Wilcoxon test. (**B**) For DLBC in the TCGA, normal tissues from the GTEx dataset were included as controls. (**C**) FAP protein expression levels in various cancers compared with normal controls. (**D**) FAP gene expression was studied according to pathological stages. ^*^P < 0.05, ^**^P < 0.01, ^***^P < 0.001.

We analyzed the mRNA expression profile of *FAP* across diverse cancer types in TCGA using TIMER2.0. As shown in Figure 1A, *FAP* was markedly elevated in tumor tissues of bladder urothelial carcinoma (BLCA), breast invasive carcinoma (BRCA), cholangiocarcinoma (CHOL), colon adenocarcinoma (COAD), esophageal carcinoma (ESCA), glioblastoma multiforme (GBM), head and neck squamous cell carcinoma (HNSC), kidney renal clear cell carcinoma (KIRC), liver hepatocellular carcinoma (LIHC), lung adenocarcinoma (LUAD), lung squamous cell carcinoma (LUSC), pheochromocytoma and paraganglioma (PCPG), prostate adenocarcinoma (PRAD), rectum adenocarcinoma (READ), stomach adenocarcinoma (STAD), and thyroid carcinoma (THCA). However, the expression level of *FAP* was found to be lower in tumor tissues of cervical squamous cell carcinoma and endocervical adenocarcinoma (CESC), kidney chromophobe (KICH), and uterine corpus endometrial carcinoma (UCEC) compared with non-tumor tissues, indicating that FAP may serve different functions in different types of cancers. Additionally, considering the lack of non-tumor tissues in certain types of cancer in TCGA datasets, we applied the normal tissues from the GTEx dataset as controls. As shown in Figure 1B, we found that *FAP* expression was significantly higher in lymphoid neoplasm diffuse large B-cell lymphoma (DLBC) compared with the normal control. Furthermore, FAP protein expression in diverse tumors and normal tissues was performed employing the CPTAC database. Results showed that the FAP protein was highly expressed in breast cancer, colon cancer, GBM, HNSC, KIRC, LIHC, LUAD and PAAD (Figure 1C).

Moreover, we investigated the association between *FAP* expression and the pathologic stages. As displayed in Figure 1D, *FAP* expression showed a general uptrend from earlier stages to later stages in BLCA, COAD, ESCA, Kidney renal papillary cell carcinoma (KIRP), Ovarian serous cystadenocarcinoma (OV), STAD, Testicular Germ Cell Tumors (TGCT) and THCA, indicating a possible promotion role of *FAP* in the tumor progression of the above-mentioned cancers.

### High levels of FAP expression predict poor survival outcomes in human cancers

**Figure 2:**
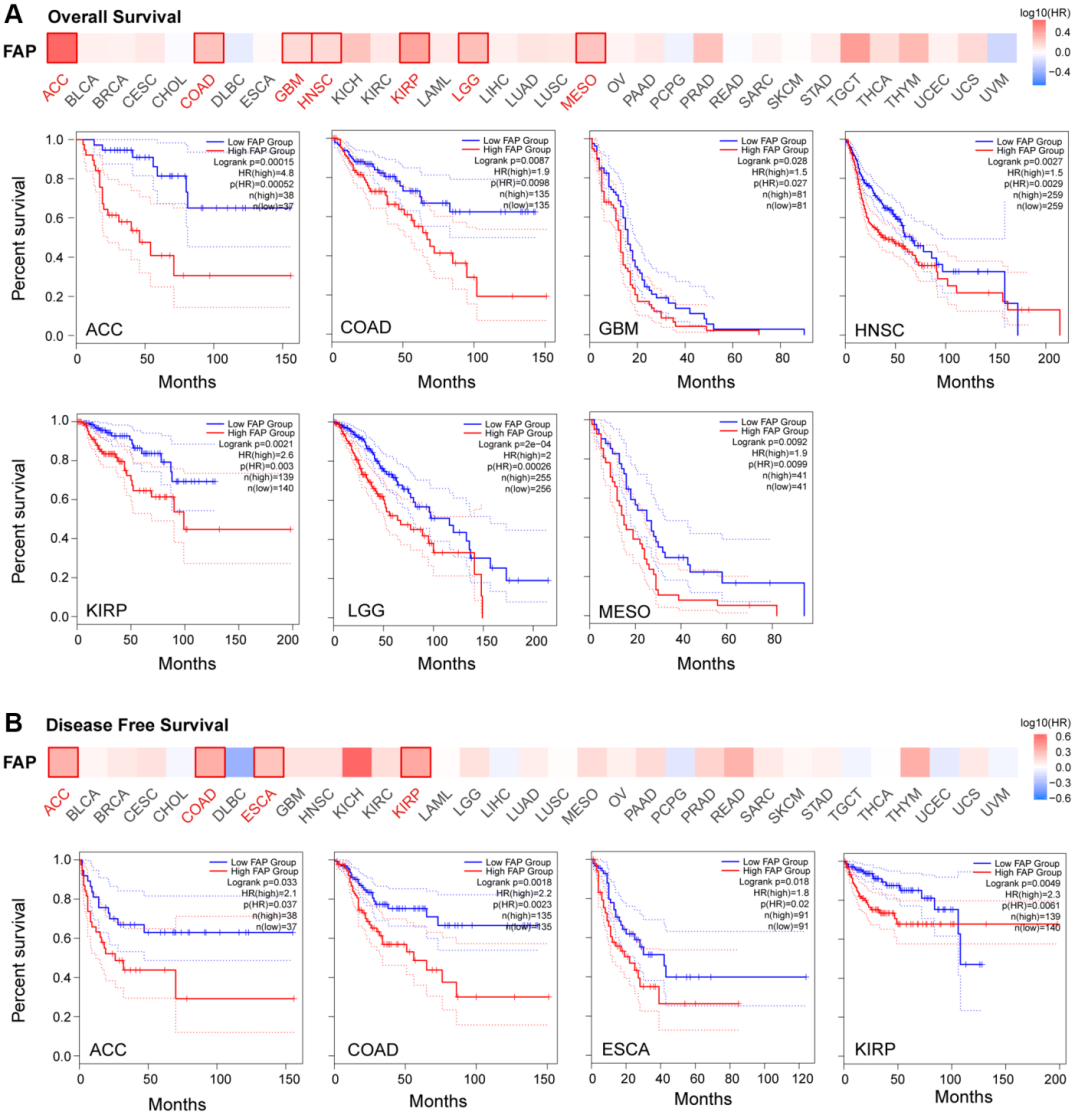
**Correlation between *FAP* gene expression and survival prognosis in TCGA cancers.** Overall Survival analysis (**A**) and Disease Free Survival analysis (**B**) were performed in diverse cancers. A cutoff value of 50% was used to split the *FAP* high-expression and low-expression cohorts. The log-rank test was used in the hypothesis.

Given the extensive expression of FAP in diverse cancers, we further investigated the correlation between *FAP* expression and clinical prognosis. Overall survival (OS) analysis showed that high expression of *FAP* was significantly correlated with poor prognosis in adrenocortical carcinoma (ACC), COAD, GBM, HNSC, KIRP, brain lower grade glioma (LGG), and Mesothelioma (MESO) (Figure 2A). Disease-Free Survival (DFS) analysis showed that high expression of *FAP* was associated with poor outcomes in ACC, COAD, ESCA, and KIRP (Figure 2B).

**Figure 3:**
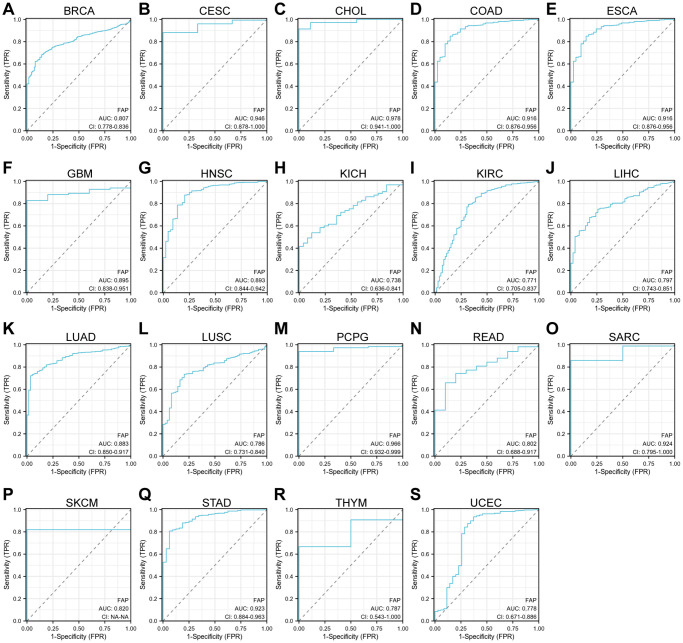
**ROC curve analyses for *FAP* in diverse cancer in TCGA database.** (**A**–**S**) ROC curve analysis and AUC values for *FAP* in BRCA, CESC, CHOL, COAD, ESCA, GBM, HNSC, KICH, KIRC, LIHC, LUAD, LUSC, PCPG, READ, SARC, SKCM, STAD, THYM, and UCEC.

Further, we assessed the diagnostic potential of FAP by plotting the receiver operating characteristic (ROC) curves. Results showed that *FAP* has a high accuracy (AUC ≥ 0.9) in predicting CESC, CHOL, COAD, ESCA, PCPG, sarcoma (SARC), and STAD. Moderate accuracy (0.9 > AUC ≥ 0.7) was shown when predicting BRCA, GBM, HNSC, KICH, KIRC, LIHC, LUAD, LUSC, READ, skin cutaneous melanoma (SKCM), thymoma (THYM), and UCEC (Figure 3). These results suggested that FAP may have the potential to act as diagnosis marker among several tumors with high sensitivity and specificity.

### Mutation feature of FAP in human cancers

**Figure 4:**
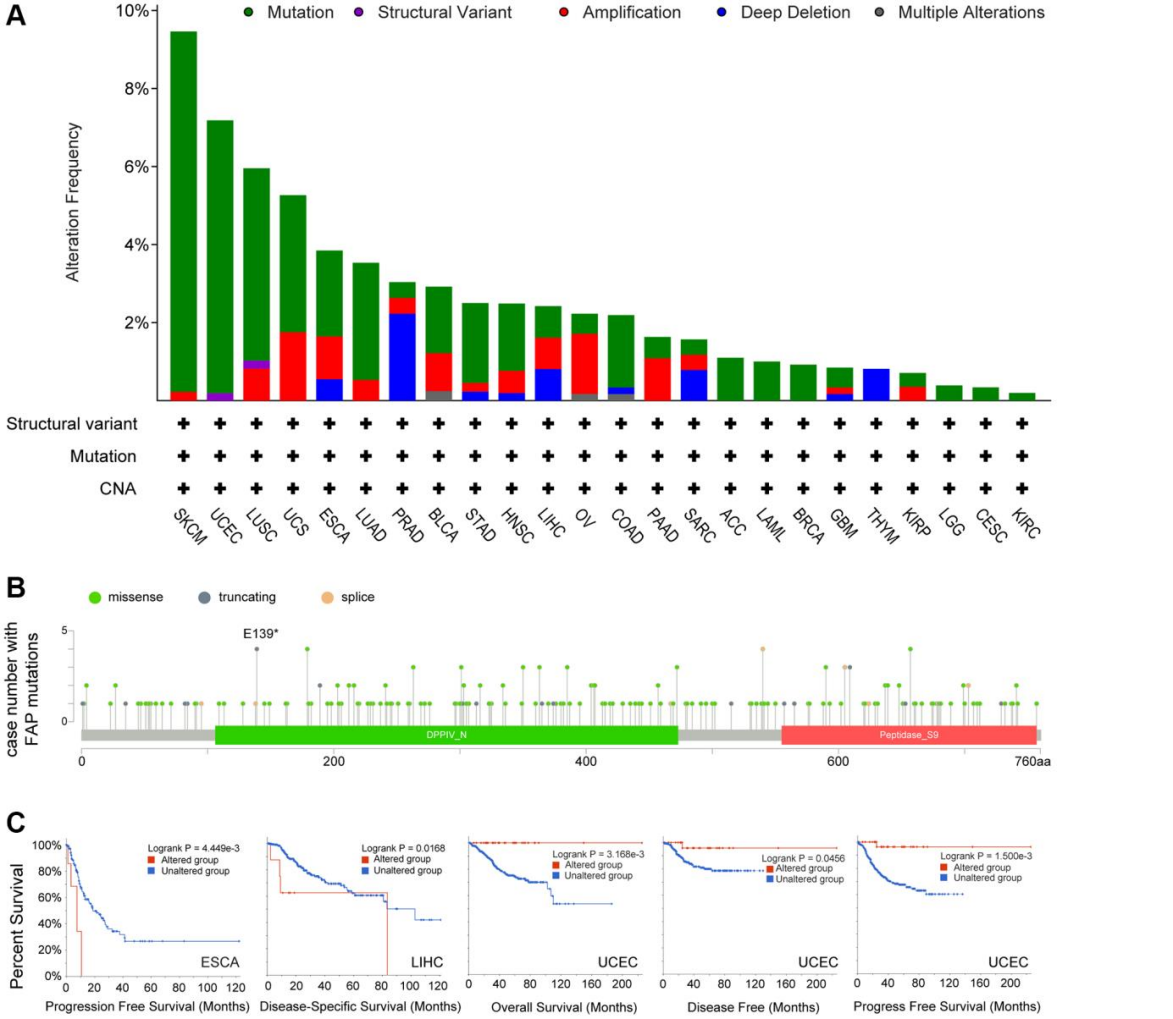
**Mutation feature of *FAP* in human cancers.** (**A**) *FAP* alteration frequency in TCGA cancer cohorts. (**B**) Sites and case numbers of *FAP* mutations were presented. (**C**) Association between genetic alteration of *FAP* and clinical survival in ESCA, LIHC, and UCEC. Kaplan-Meier plots with log-rank *p*-value were shown.

Next, we investigated the genomic alteration feature of *FAP* in different tumor samples of TCGA datasets. Results showed that mutation represented the predominant alteration, with which the alteration frequency appeared nearly 9% in SKCM, 7% in UCEC and 5% in LUSC (Figure 4A). The types, sites, and case numbers of *FAP* genetic alteration were further presented in Figure 4B. Totally 215 mutations were found, and missense was the main mutation type detected in 118 cases. Further, we accessed whether genetic alterations of *FAP* were associated with clinical outcomes. As shown in Figure 4C, the ESCA and LIHC cases with altered *FAP* showed poor progress free survival or disease specific survival respectively, while in the UCEC cases, the altered *FAP* group showed improved overall survival, disease free survival and disease specific survival compared with unaltered *FAP* group. What worth noting was that, in the UCEC cases, we observed 4 cases of E139* nonsense mutation that cannot be found in other cancer types (Figure 4B), which deserved far more investigation. Despite all this, the real role of these *FAP* gene alterations in human cancers remained uncertain, and a larger mutant sample size should be required to further explore the impact of these genetic alteration on tumor progression.

### Correlation between FAP expression and TMB and MSI

**Figure 5:**
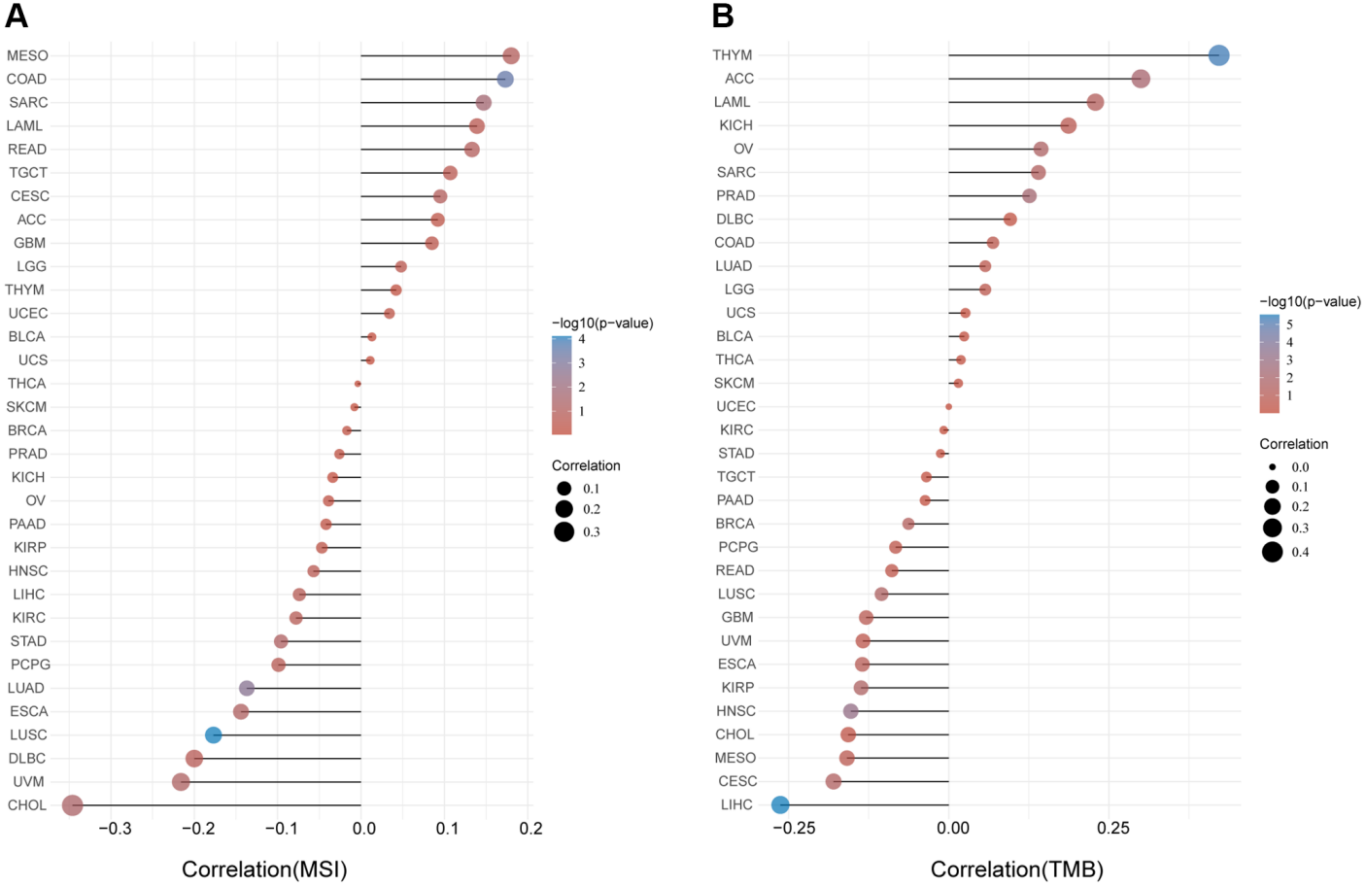
Analysis between *FAP* expression and (**A**) MSI and (**B**) TMB in human cancers with Spearman’s Correlation.

Microsatellite instability (MSI) characterizes the hypermutable state of DNA sequences caused by the lack of DNA mismatch repair activity [14]. Here we examined the correlation between FAP expression and MSI in human cancers, and found that FAP was significantly and positively correlated with COAD and SARC (Figure 5A). Tumor Mutational Burden (TMB) is the amount of DNA mutations in cancer, which appeared as a sensitive and specific biomarker in response to immune checkpoint inhibitors [15]. We explored and found a positive association between FAP and TMB in THYM, ACC, LAML, OV, SARC, and PRAD (Figure 5B). Since higher MSI or TMB may generate potent neoantigens for recognition by immune surveillance and resulting in increasing immunotherapy responses, we inferred that FAP might be available indicator in immunotherapy among several cancer types.

### FAP expression and tumor microenvironment stromal cells infiltration

**Figure 6:**
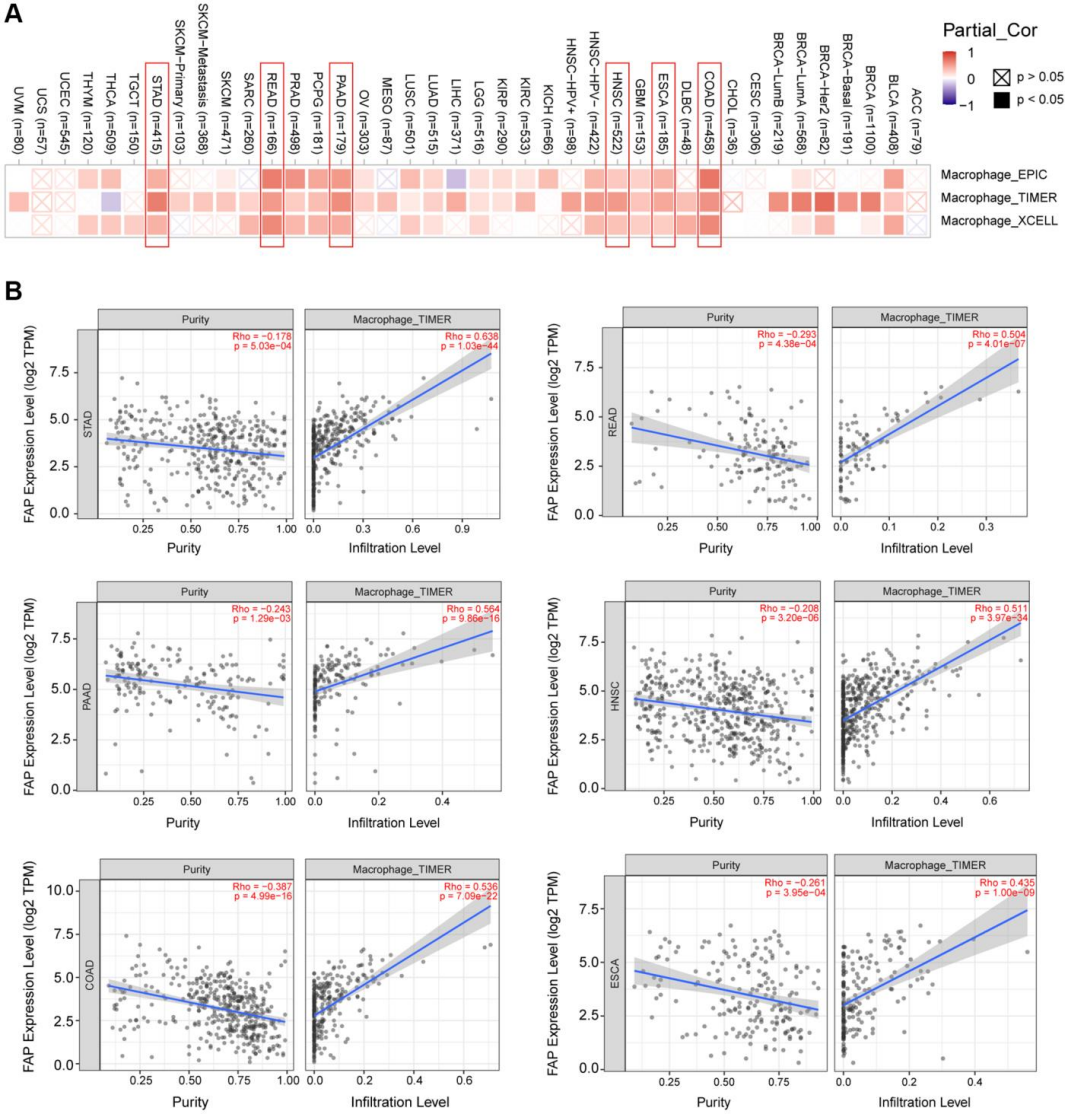
**Correlation analysis between *FAP* expression and macrophages infiltration.** (**A**) Different algorithms showed the correlation between *FAP* expression and macrophage infiltration in human cancers. (**B**) Classic scatter plotters were presented in STAD, READ, PAAD, HNSC, COAD, and ESCA. The *p*-values and Rho values were obtained via the purity-adjusted Spearman’s rank correlation test.

**Figure 7:**
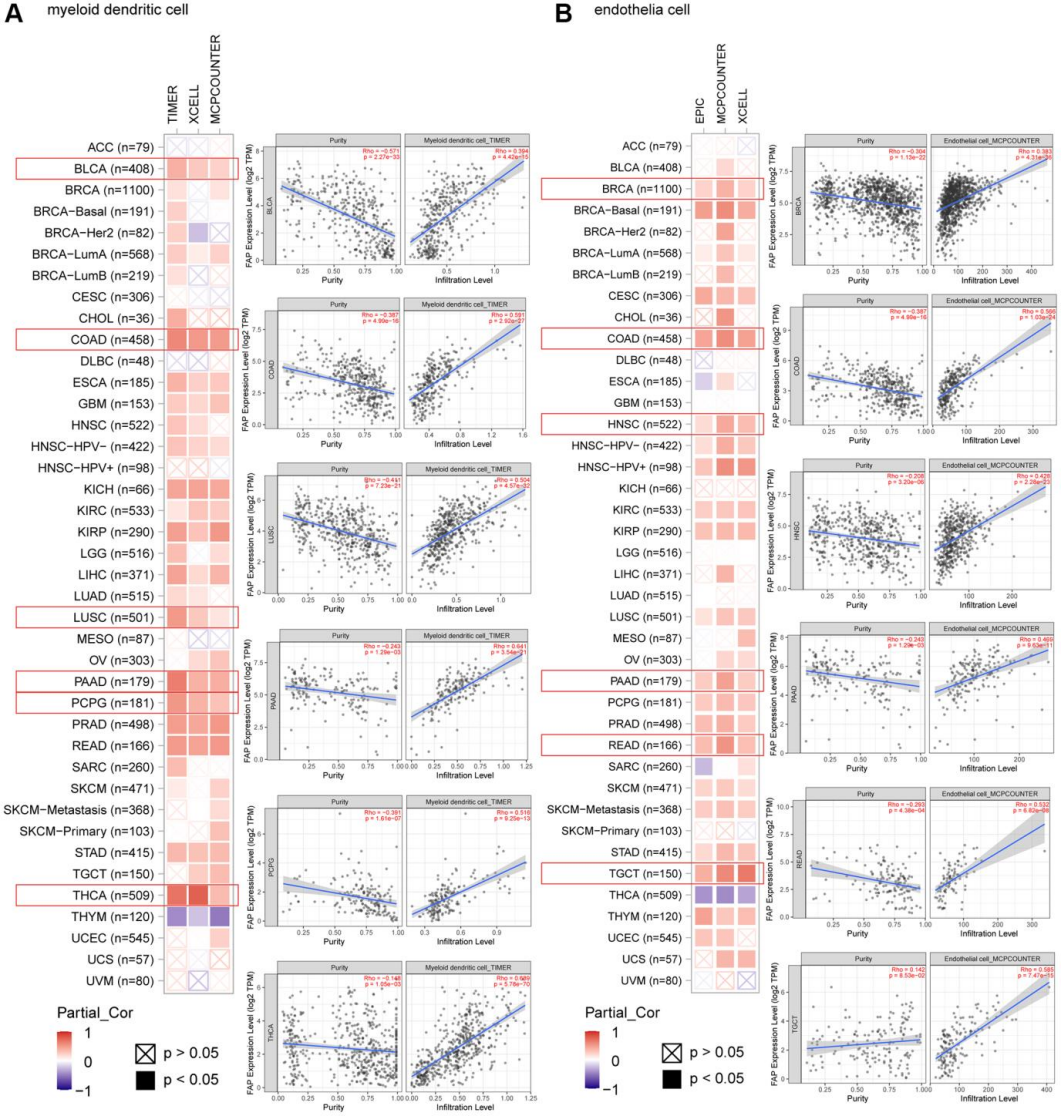
Correlation analysis between *FAP* expression and infiltration of (**A**) myeloid dendritic cells and (**B**) endothelia cells by different algorithms. Classic scatter plotters were presented.

Microenvironment stromal cells play a critical role in tumor progression and tumor resistance, to a large extent due to their interaction with cancer cells and their support of chemical signals, growth factors, and blood supply [16–18]. Herein, we investigated the relationship between *FAP* expression and infiltration of tumor microenvironment stromal cells, with the aim to decipher the role of FAP in tumor immunology and better understand the effect of these immune components with tumor development. As expected, *FAP* showed strongly positive association with cancer-associated fibroblasts (CAFs) (Supplementary Figure 1A), in line with that FAP is a universal marker of CAFs and abundantly express among CAFs [1, 19]. Surprisingly, we observed that in almost all cancer types, *FAP* expression was positively correlated with the infiltration of two major antigen-presenting cells, macrophages and myeloid dendritic cells (Figure 6 and Figure 7A). This finding showed that FAP may broadly participate in tumor immunology process, even though a cause-effect relationship could not be established in this current study. In addition, other immune cell types showed cancer-specific relation with *FAP* expression instead of universally association, and this may be due to the characteristic of diverse cancers as well as the particular roles of FAP in these cancers. Further, *FAP* was positively correlated with endothelia cells infiltration (Figure 7B), suggesting that FAP may participate in tumor progression by promoting angiogenesis. Hematopoietic stem cells (HSC) infiltration was also positively related to *FAP* expression (Supplementary Figure 1B), which may confirm the previous finding that FAP affects on HSC maintenance and is important for HSC survival and proliferation [20].

### Analysis of the relationship between FAP expression and the immunosuppressive checkpoint proteins or cytokines

**Figure 8:**
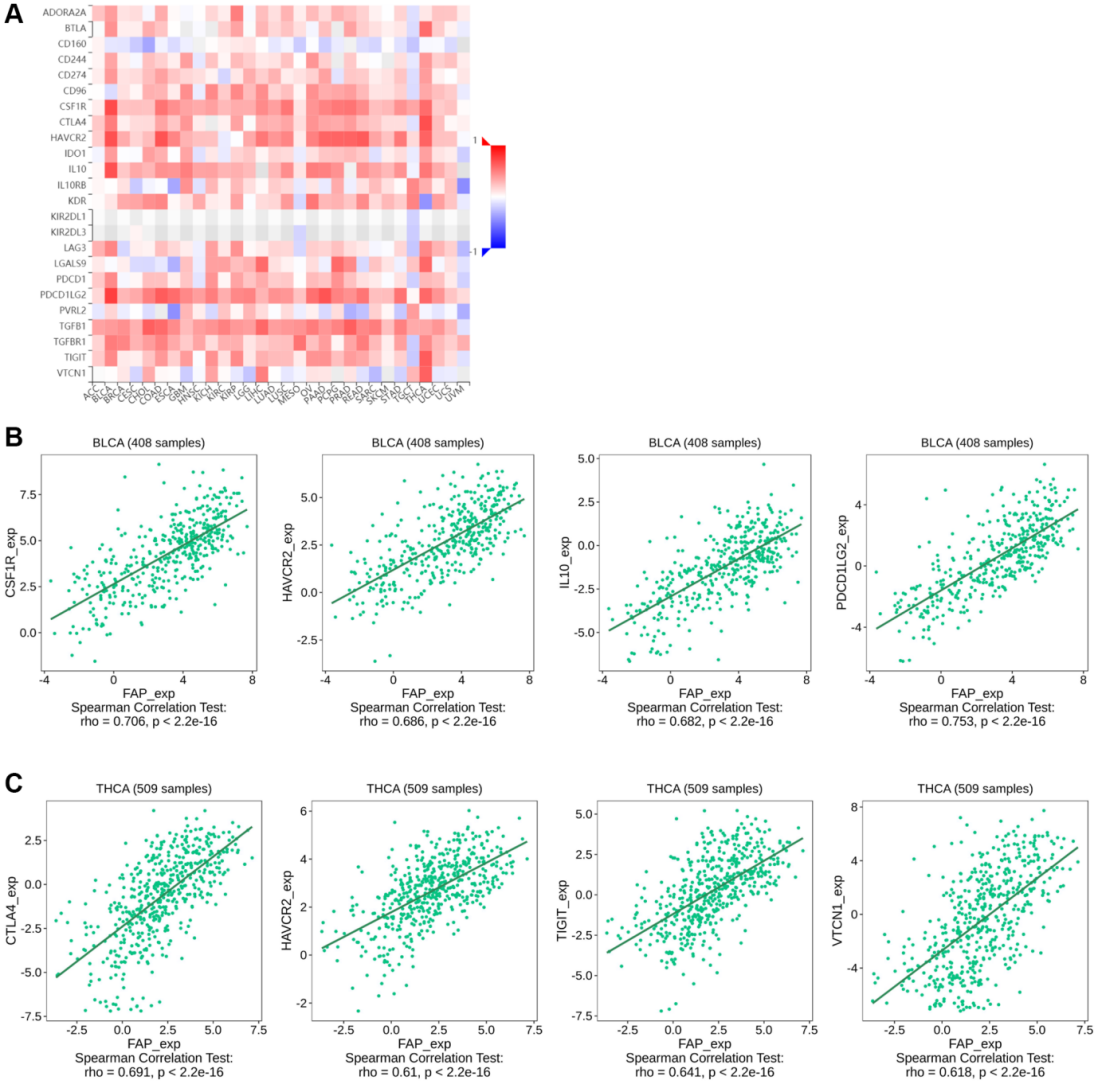
**Correlation between *FAP* expression and immunosuppressive checkpoint proteins or cytokines in human cancers.** (**A**) Correlation between *FAP* expression and immunosuppressive checkpoint proteins or cytokines was shown by the heatmap. (**B**) In BLCA, the association between *FAP* expression and CSF1R, HAVCR2, IL-10, PDCD1LG2 expression were presented. (**C**) In THCA, the association between FAP expression and CTLA4, HAVCR2, TIGIT, VTCN1 expression were presented. The Correlation between *FAP* and immune inhibitors expression were analyzed with Spearman test.

Since some of the previous studies reported that FAP may promote tumor progression by altering the intra-tumoral immune milieu and helping tumor cells escape immune surveillance [21], here we investigated the correlation between *FAP* expression and immunosuppressive signatures using the TISIDB database. According to the results, colony stimulating factor 1 receptor (CSF1R), hepatitis A virus cellular receptor 2 (HAVCR2), IL-10, PDCD1LG2, TGF-β1 as well as other immune checkpoint proteins or cytokines were almost positive correlated with *FAP* expression in different cancer types (Figure 8A), indicating that *FAP* may provide a vital help for immunosuppression. Wherein, in BLCA tumor, CSF1R (rho = 0.706), HAVCR2 (rho = 0.686), IL-10 (rho = 0.682), and PDCD1LG2 (rho = 0.753) showed the strongest positive correlation with *FAP* expression (Figure 8B). In THCA tumor, CTLA4 (rho = 0.691), HAVCR2 (rho = 0.610), TIGIT (rho = 0.641), VTCN1 (rho = 0.618) showed remarkably positive with *FAP* expression (Figure 8C). Moreover, considering FAP is a universal marker of CAFs, the heatmap results in Figure 8A suggest that FAP^+^ CAFs could maintain an immunosuppressive microenvironment by producing IL-10, IDO, TGF-β as well as other suppressive molecules reported by other researches [22].

### FAP-related gene enrichment analysis in huamn cancers

**Figure 9:**
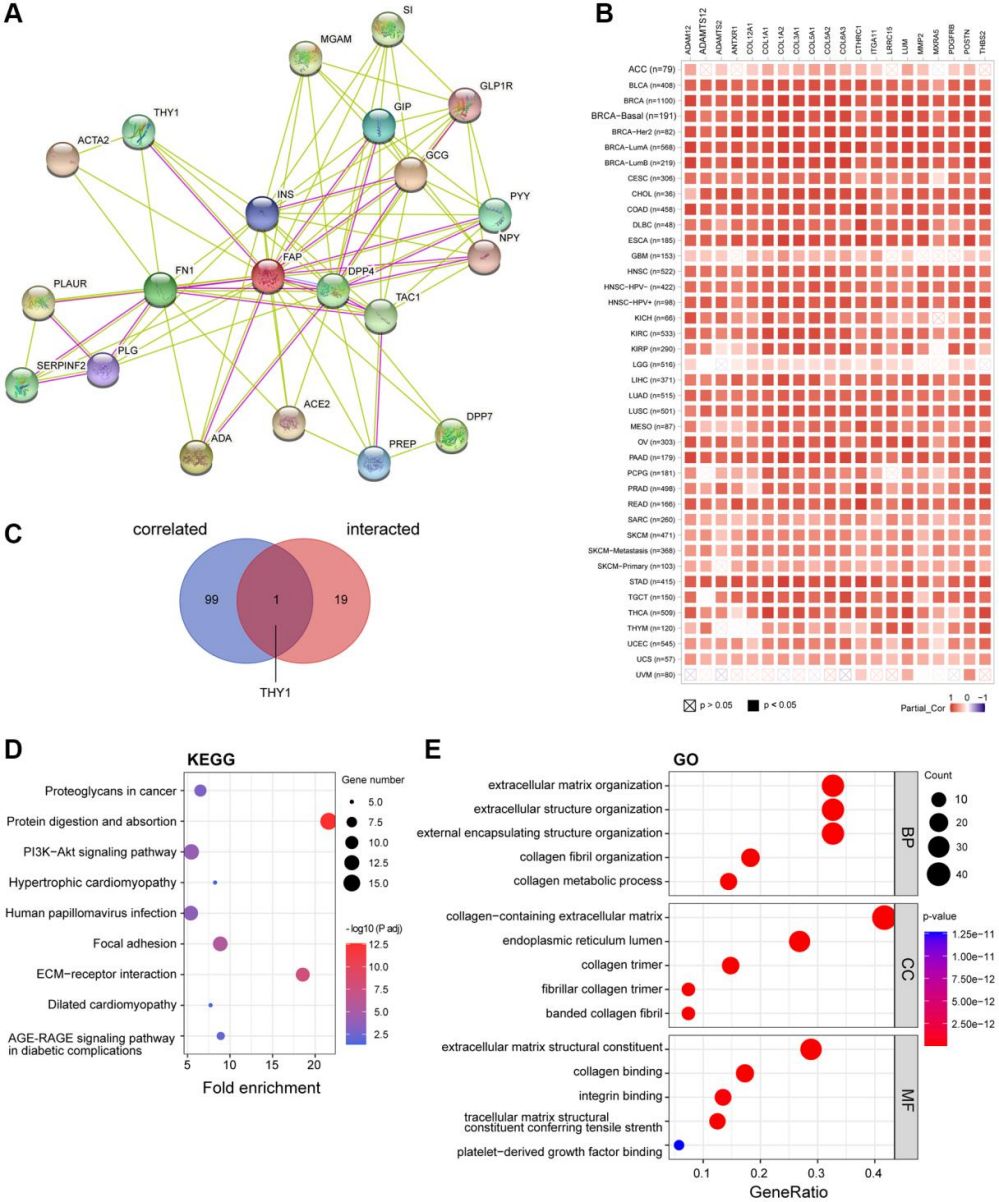
**FAP-related gene enrichment analysis.** (**A**) FAP-interacted network analyzed by STRING tool. (**B**) The heatmap displayed the top 20 of the 100 *FAP*-corelated genes in TCGA tumor database. The partial correlation (Cor) and *P*-value in the purity-adjusted Spearman’s rank correlation test were shown. (**C**) Intersection analysis of FAP-correlated and FAP-interacted partner. (**D**) KEGG enrichment analysis based on the FAP-correlated and interacted genes. (**E**) GO enrichment analysis based on the FAP-correlated and interacted genes. Abbreviations: BP: biological process; MF: molecular function; CC: cellular component.

To further study the molecular mechanism of FAP in tumorigenesis, we screened out FAP-interacted proteins and FAP-correlated genes for pathway enrichment analyses. Using the STRING online tool, we obtain a total of 20 FAP interacting proteins which were supported by experiments and text-mining evidence (Figure 9A). Then, GEPIA2 was employed to combine all tumor expression data of TCGA and obtained the top 100 genes that significantly correlated with *FAP* expression. The top 20 *FAP*-correlated genes were shown in Figure 9B. Through intersection analysis of the above-mentioned two groups, we obtained one overlapped member, Thy-1 cell surface antigen (THY1) (Figure 9C), which is also a well-known marker of fibroblast that regulates fibroblast proliferation through integrin signaling [23, 24]. Furthermore, we combined these two groups to perform KEGG and GO enrichment analyses. The KEGG pathway analysis indicated that protein digestion and absorption, ECM-receptor interaction may be significantly implicated in the tumorigenesis effect of FAP (Figure 9D). The GO analysis suggested that the majority of these genes and proteins were mainly linked to extracellular matrix and structure organization (Figure 9E).

### Prediction and construction of the ceRNA network of FAP

**Figure 10:**
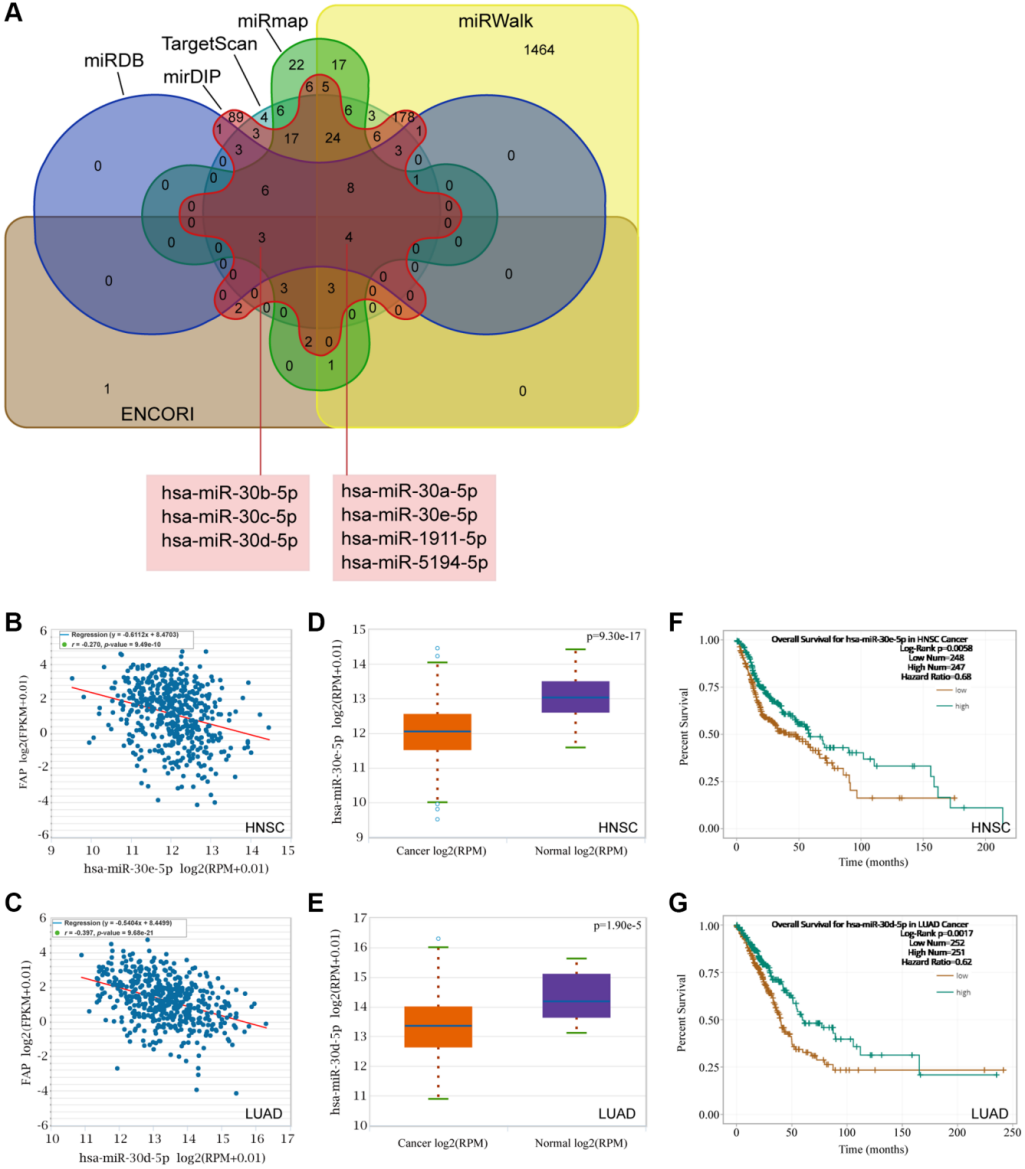
**Prediction of the miRNAs targeting FAP.** (**A**) The miRNAs targeting FAP were predicted by the ENCORI, TargetScan, miRWalk, miRmap, mirDIP, and miRDB databases and displayed by the Veen diagram. (**B**) Negative correlation of hsa-miR-30e-5p and FAP in HNSC. (**C**) Negative correlation of hsa-miR-30d-5p and FAP in LUAD. (**D**) Differential expression analysis of hsa-miR-30e-5p between HNSC and the correspond normal tissues. (**E**) Differential expression analysis of hsa-miR-30d-5p between LUAD and the correspond normal tissues. (**F**) The overall survival analysis of hsa-miR-30e-5p in HNSC. (**G**) The overall survival analysis of hsa-miR-30d-5p in LUAD.

Mounting evidence has indicated a considerable role of ceRNA networks in cancer progression. Thus, we tried to decipher the miRNAs and lncRNAs of which were able to regulate FAP expression and further affect the cancer development. Firstly, the miRNAs targeting FAP were predicted by the ENCORI, TargetScan, miRWalk, miRmap, mirDIP, and miRDB databases. The Veen diagram displayed in Figure 10A has shown that the miR-30 members, including hsa-miR-30a-5p, hsa-miR-30e-5p, as well as the hsa-miR-30b-5p, hsa-miR-30c-5p, and hsa-miR-30d-5p, were predicted in all six or at least five of the six databases, indicating that the miR-30 family may broadly participate in the regulation of FAP expression. Then, association analysis showed the inversely association between hsa-miR-30e-5p and FAP in HNSC (Figure 10B), as well as hsa-miR-30d-5p and FAP in LUAD (Figure 10C). Further, differential expression analysis revealed a relative lower level of hsa-miR-30e-5p in HNSC tumor tissues compared with the corresponded normal tissues (Figure 10D), as well a lower level of hsa-miR-30d-5p in LUAD (Figure 10E). Moreover, survival analysis demonstrated that, higher hsa-miR-30e-5p level was associated with longer overall survival in HNSC (Figure 10F), and higher hsa-miR-30d-5p level was correlated with better prognosis in LUAD (Figure 10G).

**Figure 11:**
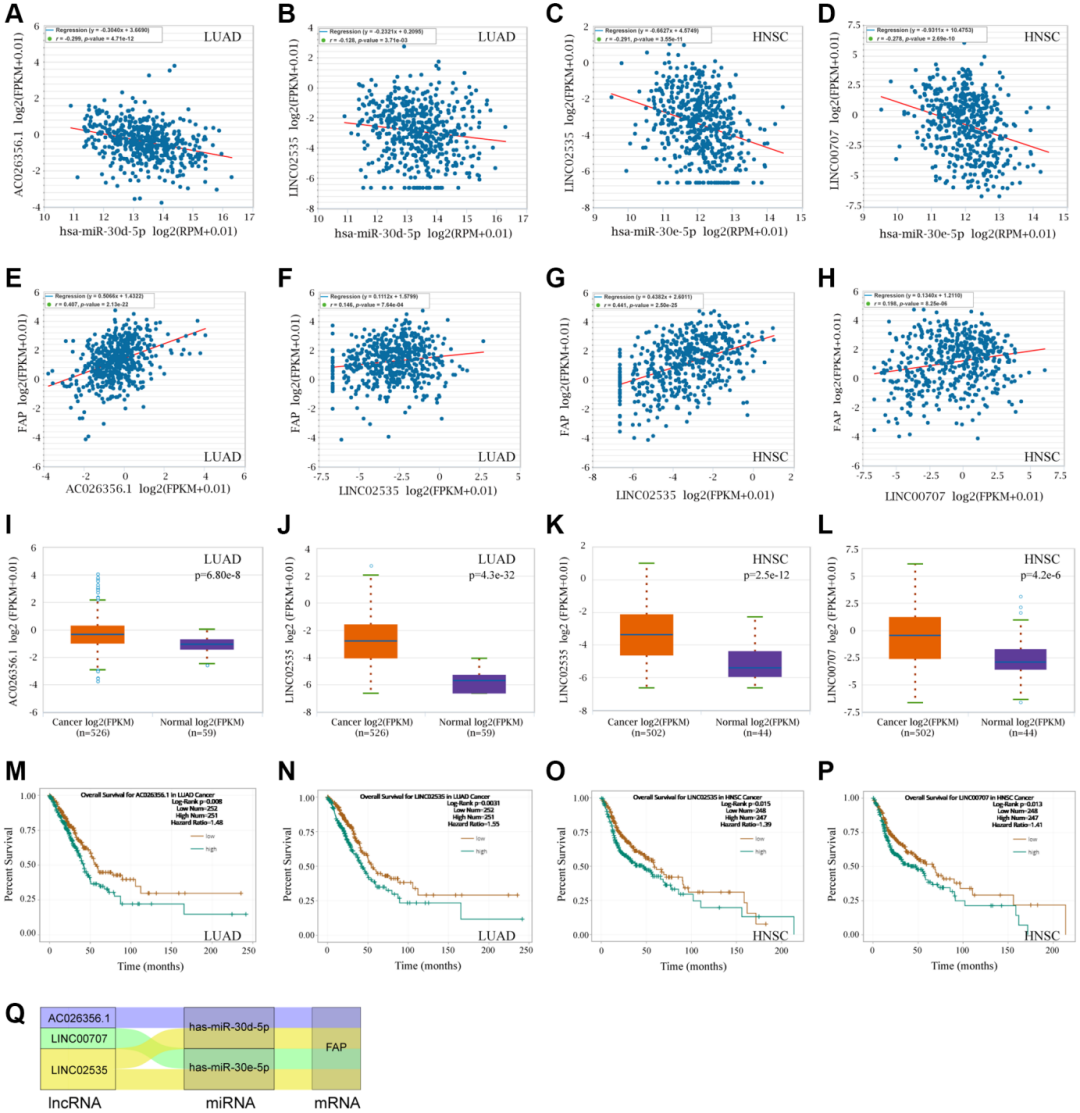
**FAP-corelated lncRNAs prediction and ceRNA network construction.** (**A**, **B**) Correlation analysis between hsa-miR-30d-5p and AC026356.1, LINC02535 in LUAD. (**C**, **D**) Correlation analysis between hsa-miR-30e-5p and LINC02535, LINC00707 in HNSC. (**E**–**H**) Correlation analysis between FAP and AC026356.1, LINC02535 in LUAD, and LINC02535, LINC00707 in HNSC. (**I**–**L**) Differential expression analysis between tumors and the correspond normal tissues of FAP and AC026356.1, LINC02535 in LUAD, and LINC02535, LINC00707 in HNSC. (**M**–**P**) The overall survival analysis of AC026356.1 in LUAD, LINC02535 in LUAD, LINC02535 in HNSC, and LINC00707 in HNSC. (**Q**) The diagram presented the ceRNA network related to FAP regulation.

Following, we used the ENCORI to search and explore the lncRNAs that may regulate the hsa-miR-30d-5p - FAP pair in LUAD, as well as the hsa-miR-30e-5p - FAP pair in HNSC. Surprisingly, we found that AC026356.1 and LINC02535 were both significantly negative with hsa-miR-30d-5p meanwhile positive with FAP in LUAD (Figure 11A, 11B, 11E, and 11F). These two lncRNAs presented higher levels in LUAD that in the correspond normal tissue (Figure 11I, 11J), and decreased overall survival rate of LUAD (Figure 11M, 11N). Similar results were observed in HNSC that LINC02535 and LINC00707 were negative with hsa-miR-30e-5p and positive with FAP in HNSC (Figure 11C, 11D, 11G and 11H). These two lncRNAs exhibited higher levels in HNSC compared with the corresponded normal tissue (Figure 11K, 11L), and decreased overall survival rate of HNSC (Figure 11O, 11P). According to the above findings, the ceRNA network was constructed and shown in Figure 11Q, which including the LINC00707/hsa-miR-30e-5p/FAP axis and LINC02535/hsa-miR-30e-5p/FAP axis in HNSC, as well as the LINC02535/hsa-miR-30d-5p/FAP axis and AC026356.1/hsa-miR-30d-5p/FAP axis in LUAD.

## DISCUSSION

Comprehensive pan-cancer analysis can provide broad insights into clinical targets and treatments, uncovering similarities and differences in cancers. In this study, we extensively explore the characteristic of FAP in pan-cancer perspective, aiming to enhance our comprehension of FAP and try to explore valid anti-tumor therapy strategies for patients. We demonstrate that FAP expression is significantly upregulated in most cancer types both in mRNA and protein levels. Higher expression of FAP is associated with advanced pathological stages in cancers including BLCA, COAD, ESCA, KIRP, OV, STAD, TGCT and THCA. The Kaplan-Meier plotter analysis further suggests an association between high *FAP* expression and poor OS or DFS in tumors including ACC, COAD, GBM, HNSC, KIRP, LGG, MESO, and ESCA. ROC analysis also indicates a moderate to high diagnostic capability of FAP for most cancers. Consistent with our previous research [25], these above findings further support that FAP may serve as a biomarker and therapeutic target for tumor treatment. Additionally, we also find that genetic alteration of *FAP* is linked to the clinical outcome of ESCA, LIHC and UCEC. MSI and TMB analysis reveal a positive correlation between *FAP* and COAD, SARC, THYM, ACC, LAML, OV, and PRAD. These observations are valuable for further research to exploit more prognostic factors.

The mechanism that aberrant FAP expression promotes tumor progression probably lies in two main hypotheses [1, 26]. The first hypothesis is that FAP regulates extracellular matrix (ECM) remodeling and reorganization by cleaving substrate peptides or proteins, resulting in promoting tumor cells migration and invasion. As evidenced by KGGG and GO gene enrichment analysis in this study, we confirmed that FAP mainly directed ECM-receptor interaction as well as extracellular matrix and structure process in integrin-dependent pathways. Another hypothesis lies in that aberrant FAP expression triggers abnormal intracellular signaling pathways. We observed that FAP was strongly linked to the PI3K-AKT signaling pathway in this study. In other studies, FAP was also reported as an activate regulator in Ras-ERK pathway, TGF-β signaling, SHH/Glil signaling, as well as Wnt/β-catenin pathway [1, 5, 27].

The ceRNAs represent a novel pattern of gene regulation that acts a vital role in tumor occurrence and development. Recently, a few miRNAs and lncRNAs with the capacity to regulate FAP have been reported. MiR-30a targeted and inhibited FAP expression, thus resulting in the suppression of oral cancer or gastric cancer [28, 29]. AC007099.1/has-miR-7152/FAP axis was associated with LIHC progression [30]. SNHG16/hsa-miRNA-30c-5p/FAP axis was constructed in STAD, however, neither significant association between FAP and SNHG16, nor prolonged survival of SNHG16 was observed [12]. In this study, based on the ceRNA hypothesis, we hunted for miRNAs and lncRNAs to construct the ceRNA network that may regulate the FAP expression. We surprisingly identified the inhibitory effect of hsa-miR-30e-5p in LUAD, as well as hsa-miR-30d-5p in LUAD, which provided new evidence to support the tumor suppressor role of the miR-30 family [31]. To our knowledge, it is for the first time that LINC00707/hsa-miR-30e-5p/FAP axis and LINC02535/hsa-miR-30e-5p/FAP axis in HNSC, as well as the LINC02535/hsa-miR-30d-5p/FAP axis and AC026356.1/hsa-miR-30d-5p/FAP axis in LUAD were successfully constructed and demonstrated. Our new-found ceRNA network provided a better understanding of the molecular mechanisms of FAP regulation, and indicated that FAP as well as the correlated miRNAs or lncRNAs may be promising targets or biomarkers in cancer therapy. In the future, further experimental studies should be performed to verify our findings in this research.

Over the past decades, immunotherapy has revolutionized cancer treatment by providing robust options for numerous cancers that were previously unmanageable. Unfortunately, there are still several tumors responded poorly to immunotherapy and characterized by high mortality [32]. Thus, in this study, we explore the immunological features of FAP in multiple cancers, aiming to find clues for overcoming the tumor immunotherapy barriers. We surprisingly find that *FAP* expression level is remarkably related to the infiltration of tumor-associated fibroblasts, macrophages, myeloid dendritic cells, endothelia cells as well as hematopoietic stem cells in most types of cancers. Moreover, we also find that *FAP* expression is positively associated with various immunosuppressive checkpoint proteins or cytokines including CSF1R, PDCD1LG2, IL-10 and IDO. Herein, we briefly discuss the immunosuppressive effect of FAP found in this study as well as in other researches.

FAP-positive cancer associated fibroblasts (FAP^+^ CAFs) recruit macrophage infiltration into the tumor microenvironment and thus shape the immune suppression landscape. In this study, we found that FAP expression was positively correlated with the infiltration of cancer associated fibroblasts, macrophages, as well as CSF1R protein in most cancers. Considering as an essential source of CSF1, we inferred that fibroblasts may be recognized by CSF1R that mainly expressed on the macrophages, thus subsequently recruiting the macrophages. Fibroblast-macrophage interaction that centers around the CSF1-CSF1R signaling axis has already been proved essential in the occurrence and progression of various cancers [33, 34]. Moreover, Qi et al. found that FAP^+^ CAFs and SPP1^+^ (secreted phosphoprotein 1) macrophages interacted with each other through chemerin, TGF-β, and IL-1, accompanied by stimulating the formation of immune-excluded desmoplastic structure and limiting the T cell infiltration [35]. Anna et al. found that FAP^+^ CAFs promote the recruitment of monocytes by mediating the cleavage of collagen which in turn creates an adhesion substrate for class A scavenger receptor (SR-A) expressed at the surface of macrophages [36]. By secreting CCL-2, IL-6, and CXCL8, FAP^+^ CAF-like cells are able to induce macrophages differentiation into tumor-associated macrophages [37]. Tumor-associated macrophages not only lacked the capacity to present antigen, but also suppressed the Th1-adaptive antitumor immune responses [32, 38].

Myeloid dendritic cell is another kind of antigen-presenting cells that we found to be positively correlated with *FAP* expression among various cancers in this research. Dendritic cell was usually thought to actively induce immune responses. However, recently increasing evidences indicated that dendritic cells recruited to the tumor microenvironment undergo changes that endow them with regulatory functions favorable for tumor progression. FAP positively related IL-10, IDO and TGF-β that demonstrated in Figure 7A, as well as VEGF, IL-6, M-CSF, COX2, PGE2, gangliosides and other suppressive molecules enriched in the tumor microenvironment as reported by other studies, can block dendritic cells differentiation and maturation. These immature or partially differentiated myeloid dendritic cells have proven to further induce either suppressive Tregs or T-cell unresponsiveness [22, 39, 40], resulting in forming one of the crucial compositions in the immunosuppressive network within the tumor microenvironment.

In addition to macrophages and myeloid dendritic cells, FAP can also block the anti-tumor response through regulating T cell attraction, differentiation, and retention through various immunosuppressive checkpoint proteins and cytokines. FAP^+^ CAFs attract CD4^+^CD25^+^ T cells by CXCL12 and retain them by OX40L and PD-L2, which is highly expressed in FAP^+^ CAFs as observed in this study and other reports. Subsequently, FAP^+^ CAFs promote their differentiation into Tregs and survival, thus promote an immunosuppressive microenvironment [21, 41, 42]. Other studies found that, by releasing CXCL12, CCL5, lactate or other signals, FAP^+^ CAFs recruit Tregs or shape the T-cell polarization into Tregs [43–45]. Moreover, FAP can also induce high expression of immunosuppressive checkpoint proteins at the surface of T cells. In breast cancer, FAP^+^ CAFs particularly the ECM-myCAF and TGFβ-myCAF clusters, increase PD-1 and CTLA-4 expression on the surface of CD4^+^CD25^+^FOXP3^+^ Tregs [21, 46]. In melanoma, FAP^+^ CAFs induced TIGIT and BTLA expression on cytotoxic T lymphocytes via increased arginase activity [47]. In regard to MDSCs, FAP^+^ CAFs releasing CCL2 is recognized by the CCL2 receptor that expressed on circulating MDSCs, leading to the poly-morphonuclear MDSCs infiltration or circulating MDSCs recruitment into the tumor microenvironment [48–50]. These above findings may indicate the benefit of combining anti-FAP treatment and immune blockade treatment for anti-tumor therapy and strategies.

Tumor progression is often accompanied and supported by the ingrowth of blood vessels, consistent with the requirement to access to the circulation system for thrive. In this study, we observed that FAP expression is positively correlated with endothelial cell infiltration in almost cancer types, indicating that FAP may also exert its tumor progression role by promoting angiogenesis in a wide range of cancer types. Previously studies have validated that high expression of FAP increased microvascular density. FAP^+^ mesenchymal cells express proangiogenic factors including VEGF and angiopoietin, thus recruiting endothelial cells, promote microvascular formation and supply of oxygen and nutrients to the tumors [1, 51, 52]. In turn, endothelial cells secrete factors that support the survival and self-renewal of the cancer cells. Additional studies have also identified matrix metallopeptidase 9 (MMP9) is responsible for angiogenic phenotypes of FAP expressing tumors, since this FAP co-expressed protein MMP9 is a known pro-angiogenic signal [53].

In conclusion, our research reveals novel insights and extracts the potential antitumor values of FAP from a pan-cancer perspective, particularly in the area of immunotherapy. We demonstrate that FAP is upregulated in various cancer types, and increased FAP expression is associated with advanced pathological stages, poor prognosis and immunosuppressive tumor microenvironment. Particular ceRNA network was found to regulate the FAP expression. The effect of FAP on tumor progression, immunosuppression and angiogenesis could be a consequence of its extracellular matrix remodeling capability as well as its activation of intracellular signaling. Further investigations are warranted to identify novel strategies targeting FAP to overcome tumor stromal barriers and broaden the clinical effectiveness of immunotherapy.

## MATERIALS AND METHODS

### Gene and protein expression analysis

We used the Tumor Immune Estimation Resource (TIMER, http://timer.cistrome.org) to analyze the expression level of *FAP* between tumor and non-tumor tissues in diverse types of cancers derived from the TCGA project. For certain tumors without non-tumor tissues in TCGA, the Gene Expression Profiling Interactive Analysis (GEPIA2, http://gepia2.cancer-pku.cn) was used to examine the variation of *FAP* expression by matching TCGA and Genotype-Tissue Expression (GTEx) datasets. Besides, *FAP* expression in distinct pathological stages in TCGA tumor was analyzed by GEPIA2 as well. Furthermore, the Clinical Proteomic Tumor Analysis Consortium (CPTAC) module of UALCAN (http://ualcan.path.uab.edu/analysis-prot.html) was used to analyze the FAP protein expression across tumor and normal samples.

### Survival analysis

The “Survival Analysis” module in GEPIA2 was used to analyze the relationship between *FAP* expression and the survival prognosis of TCGA cancers.

### Receiver operating characteristic (ROC) curve analysis

Data was downloaded from the TCGA databases. The diagnostic values were calculated using the R package “pROC” and visualized by the “ggplot2”.

### Genetic alteration analysis

The cBioPortal (https://www.cbioportal.org) was used to analyze the genetic alteration of *FAP* in TCGA cancers. The outcomes of alteration frequency, mutation type, and copy number alteration (CNA) were presented in the “cancer types summary” module. The survival differences with or without genetic alteration were observed in the “Comparison/ Survival” module.

### Microsatellite instability (MSI) and tumor mutational burden (TMB) analysis

RNA-sequencing expression profiles and corresponding clinical information were downloaded from the TCGA database. Correlations between *FAP* expression and the MSI and TMB were analyzed using R 4.0.3 software with Spearman’s correlation.

### Tumor microenvironment stromal cells infiltration analysis

The “Immune-Gene” module of the TIMER 2.0 was used to analyze the correlation of *FAP* expression and tumor microenvironment stromal cells infiltration across TCGA tumors. The EPIC, TIMER, XCELL, MCPCOUNTER, and TIDE algorithms were applied for infiltration estimations. Additionally, TISIDB (http://cis.hku.hk/TISIDB) was used to analyze the association between *FAP* and immunosuppressive checkpoint proteins or cytokines.

### FAP-related gene enrichment analysis

“Similar Gene Detection” module of GEPIA2 was used to obtain *FAP*-related genes based on TCGA datasets, and “Gene Corr” module of TIMER2.0 was utilized to collect the heatmap data of the aforementioned genes. The STRING (https://string-db.org) was used to obtain proteins that interacted with FAP. We then combined the above FAP-related genes and *FAP*-interacted genes to perform KEGG (Kyoto encyclopedia of genes and genomes) and GO (Gene Ontology) enrichment analysis through the Database for Annotation, Visualization, and Integrated Discovery (DAVID, https://david.ncifcrf.gov) website tool. Enrichment analysis was finally conducted and visualized by the R software package “clusterProfiler” and “ggplot2”.

### Construction of the competing endogenous RNA (ceRNA) network

The miRNAs targeting FAP were predicted and studied by the ENCORI (https://starbase.sysu.edu.cn/), TargetScan (https://mirmap.ezlab.org/app/), miRWalk (http://129.206.7.150/), miRmap (https://mirmap.ezlab.org/app/), mirDIP (http://ophid.utoronto.ca/mirDIP/index.jsp), and miRDB (http://www.mirdb.org/) databases, respectively. The upstream lncRNAs were predicted and studied by the ENCORI as well.

## Supplementary Materials

Supplementary Figure 1
