# Supplementary material for: Comprehensive analysis of the oncogenic and immunological role of FAP and identification of the ceRNA network in human cancers
Source: Aging (Albany NY). 2023 May 9;15(9):3738–58. doi: 10.18632/aging.204707 (PMC10449273; doi:10.18632/aging.204707)
Supplement: Supplementary Figure 1 [file aging-15-204707-s001.pdf]

SUPPLEMENTARY FIGURE

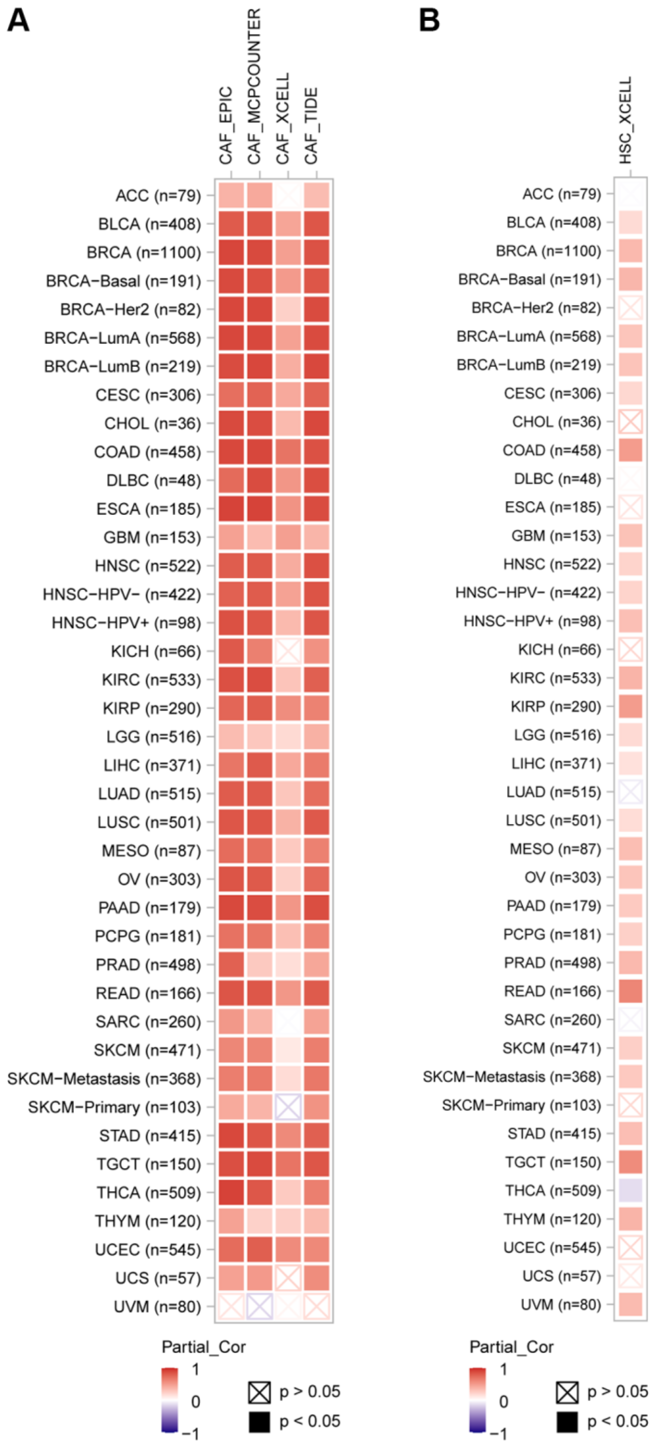

Supplementary Figure 1. Different algorithms showed positively correlation between *FAP* expression and (**A**) Cancer-Associated Fibroblast infiltration and (**B**) hematopoietic stem cells in TCGA datasets.
